# Methylome analysis for spina bifida shows *SOX18* hypomethylation as a risk factor with evidence for a complex (epi)genetic interplay to affect neural tube development

**DOI:** 10.1186/s13148-016-0272-8

**Published:** 2016-10-13

**Authors:** Anne Rochtus, Raf Winand, Griet Laenen, Elise Vangeel, Benedetta Izzi, Christine Wittevrongel, Yves Moreau, Carla Verpoorten, Katrien Jansen, Chris Van Geet, Kathleen Freson

**Affiliations:** 1 Department of Cardiovascular Sciences, Center for Molecular and Vascular Biology, University of Leuven, Campus Gasthuisberg, O&N1, Herestraat 49, Box 911, 3000 Leuven, Belgium; 2 Department of Pediatrics, University Hospitals Leuven, Leuven, Belgium; 3 Department of Electrical Engineering ESAT-SCD, University of Leuven, Leuven, Belgium; 4 Genetic Research About Stress and Psychiatry (GRASP), University of Leuven, Leuven, Belgium

**Keywords:** Neural tube defects, Myelomeningocele, Spina bifida, DNA methylation, Epigenetics, *SOX18*, *BMP4*

## Abstract

**Background:**

Neural tube defects (NTDs) are severe congenital malformations that arise from failure of neurulation during early embryonic development. The molecular basis underlying most human NTDs still remains largely unknown. Based on the hypothesis that folic acid prevents NTDs by stimulating methylation reactions, DNA methylation changes could play a role in NTDs. We performed a methylome analysis for patients with myelomeningocele (MMC). Using a candidate CpG analysis for *HOX* genes, a significant association between *HOXB7* hypomethylation and MMC was found.

**Methods:**

In the current study, we analyzed leukocyte methylome data of ten patients with MMC and six controls using Illumina Methylation Analyzer and WateRmelon R-packages and performed validation studies using larger MMC and control cohorts with Sequenom EpiTYPER.

**Results:**

The methylome analysis showed 75 CpGs in 45 genes that are significantly differentially methylated in MMC patients. CpG-specific methylation differences were next replicated for the top six candidate genes *ABAT*, *CNTNAP1*, *SLC1A6*, *SNED1*, *SOX18*, and *TEPP* but only for the *SOX18* locus a significant overall hypomethylation was observed (*P* value = 0.0003). Chemically induced DNA demethylation in HEK cells resulted in *SOX18* hypomethylation and increased expression. Injection of *sox18* mRNA in zebrafish resulted in abnormal neural tube formation. Quantification of DNA methylation for the *SOX18* locus was also determined for five families where parents had normal methylation values compared to significant lower values for both the MMC as their non-affected child. *SOX18* methylation studies were performed for a MMC patient with a paternally inherited chromosomal deletion that includes *BMP4*. The patient showed extreme *SOX18* hypomethylation similar to his healthy mother while his father had normal methylation values.

**Conclusions:**

This is the first genome-wide methylation study in leukocytes for patients with NTDs. We report *SOX18* as a novel MMC risk gene but our findings also suggest that *SOX18* hypomethylation must interplay with environmental and (epi)genetic factors to cause NTDs. Further studies are needed that combine methylome data with next-generation sequencing approaches to unravel NTD etiology.

**Electronic supplementary material:**

The online version of this article (doi:10.1186/s13148-016-0272-8) contains supplementary material, which is available to authorized users.

## Background

Neural tube defects (NTDs) are severe congenital malformations with a frequency of one to two per 1000 pregnancies [1]. Despite its high prevalence and severe consequences, the underlying molecular basis of most human NTDs remains largely unknown. Folic acid supplementation is known to reduce the incidence of NTDs, though its mode of action for NTD prevention is poorly understood. The only well-characterized genetic risk factor for human NTDs is the 677C>T change in the 5,10-methylene tetrahydrofolate reductase (*MTHFR*) gene. This variant is known to lead to hyperhomocysteinemia and global DNA hypomethylation [2–4]. This association led to the hypothesis that folic acid prevents NTDs by stimulating DNA methylation [2, 5]. To investigate whether changes in DNA methylation are involved in NTDs, different research groups have quantified global (e.g., via LINE-1 elements) or locus-specific (e.g., imprinted genes, transposable elements, DNA repair enzymes) DNA methylation for patients with NTDs using DNA from diverse types of tissues [5]. Methylation studies of imprinted genes in post-mortem brain tissue of NTD patients showed significant hypermethylation of the *IGF2* and *H19* differentially methylated regions (DMRs) [6, 7]. Other studies focused on the analysis of DNA repair genes, but only *MGMT* was found to be slightly hypomethylated in brain tissue of NTD patients [8]. Investigation of DNA methylation of the candidate genes folate receptor α (*FOLR1*), proton-coupled folate transporter (*PCFT*), and reduced folate carrier 1 (*RFC1*) genes did not show significantly differences between patients and controls, though some minor differences were observed according to *RFC1* 80G>A genotype [9]. This suggests a gene-nutrition interaction between folate intake and the *RFC1* genotype in NTD-affected births. The most robust finding of all DNA methylation studies for NTDs was found for LINE-1 and global DNA methylation. Lower levels of LINE-1 and global DNA methylation were found for NTD patients. The decrease in methylation was even more pronounced for cranial compared to caudal NTDs [10]. Previously, we performed a genome-wide DNA methylation study using the HumanMethylation 450K BeadChip (HM450k) and leukocyte DNA from ten patients with myelomeningocele (MMC) and six unrelated healthy controls. We analyzed these data using a candidate-gene approach for the Homeobox (*HOX*) genes [11]. *HOX* genes play a central role in neural tube development and are regulated in a spatiotemporal and collinear manner, partly by epigenetic modifications [12]. We found evidence that *HOXB7* hypomethylation is a potential risk factor for MMC. Interestingly, a study by Kok et al. found that DNA methylation of *HOXB7* in particular but also of the majority of the other *HOX* genes tend to be increased after folic acid and vitamin B12 supplementation [13]. A recent meta-analysis investigated the impact of maternal plasma folate during pregnancy on DNA methylation in cord blood [14]. They found that multiple developmental processes are influenced by maternal folate, including neural tube development.

For this study, we analyzed the data of the genome-wide DNA methylation study without focusing on candidate genes or pathways to discover novel genes with methylation changes associated to NTDs. The analysis was performed using Illumina Methylation Analyzer (IMA) [15] and WateRmelon [16] R-packages. Findings were confirmed using a locus-specific validation study with the Sequenom EpiTYPER and in larger MMC and control cohorts. The most significant overall hypomethylation was found for the *SOX18* locus in MMC patients. Furthermore, *SOX18* expression studies were performed in chemically induced hypomethylated DNA from HEK cells, and neural tube development was studied in *sox18* messenger RNA (mRNA)-injected zebrafish embryos. Additionally, we quantified *SOX18* methylation in five families that include parents, the MMC patient, and its non-affected sibling and in one family with a MMC patient that has a paternally inherited *BMP4* deletion.

## Results

### DNA methylation of LINE elements and folic acid regulatory genes

Findings of global DNA and LINE-1 hypomethylation in patients with NTDs [10, 17] suggest that genomic instability might interfere with neural tube closure. Analysis of LINE-1 methylation in our HM450k study showed no overall methylation difference between MMC patients and controls (mean *β* value was 77.6 versus 77.7 %; respectively). However, unsupervised hierarchical clustering analysis grouped almost all MMC patients separately from the controls, which suggests a similar DNA methylation pattern (Additional file 1: Figure S1; Additional file 5: Table S1).

We next analyzed 43 genes involved in the folic acid and the one carbon metabolism [18], but there were no significant methylation differences between MMC patients and controls (Additional file 2: Figure S2; Additional file 5: Table S2). According to unsupervised hierarchical clustering analysis, samples are clustered irrespective of the subgroup.

### Methylome analysis for gene identification

**Fig. 1:**
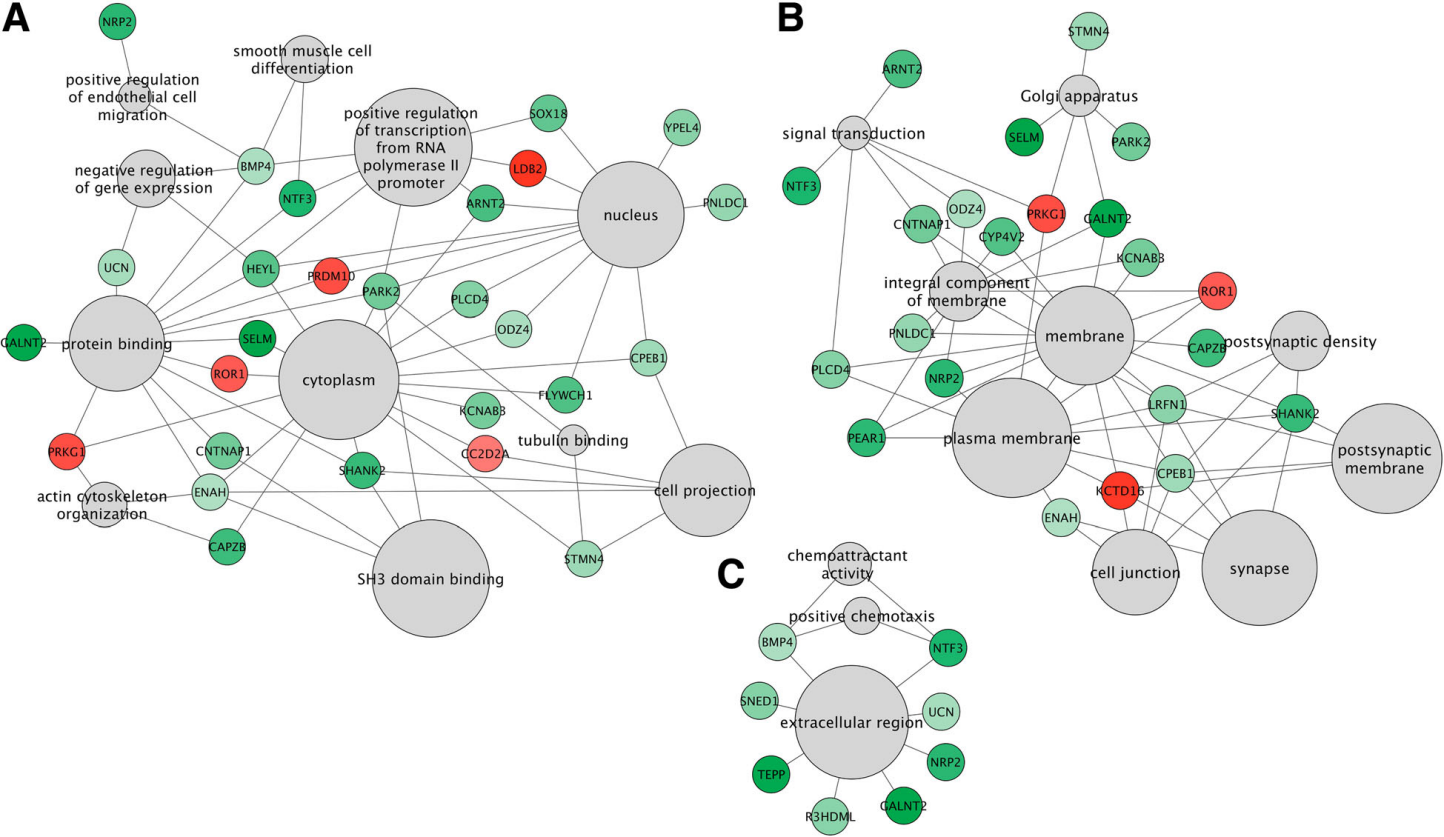
Gene ontology enrichment map based on top differentially methylated genes in MMC patients. The map displays three clusters of functionally related gene-sets in MMC patients versus controls (**a**–**c**). *Gray nodes* represent GO classes and the size of each node is correlated with its significance (adjusted *P* value). *Green* and *red* nodes represent hypomethylated and hypermethylated genes, respectively. The intensity of the color is correlated with *β* difference

The analysis of HM450k data showed significant methylation differences for 45 genes (comprising 75 CpG sites) using selection criteria that include a *β* value >0.10 and *P* value <0.01. Out of these 75 CpG sites, 72 % CpG sites were associated with hypomethylation for MMC patients compared to controls (Additional file 5: Table S3). Further selection of genes with more than one single significantly differentially methylated CpG resulted in six candidate genes *ABAT*, *CNTNAP1*, *SLC1A6*, *SNED1*, *SOX18*, and *TEPP* (Table 1). Pathway analysis of the 45 genes showed enrichment of fundamental developmental pathways. The gene ontology classes implicated include cytoplasm, transcription, neuron projection, synaptic processes, cell projection, and some minor others (Fig. 1). Interestingly, our candidate genes seem to act together through various pathways, as they are often involved in divergent gene ontology categories (Additional file 5: Table S4). This pathway analysis favors the hypothesis that NTDs are the result of a complex multifactorial combination. Results for the most significant CpGs according to only having a *P* value <0.001 (independent of the change in *β* value) are represented in Additional file 5: Table S5. Among these 370 highly significant CpGs, 58 % are hypomethylated in patients with NTDs.

**Table 1:** Top six differentially methylated genes investigated by HM450k and selected for validation with Sequenom EpiTYPER

Gene	Illumina ID	Chr	Mapinfo	WateRmelon		IMA R-package		Gene group
				*P* value	*β* diff	*P* value	*β* diff	
ABAT Chr16p13	cg01881182	16	8806531	0.0017	−0.20	0.0047	−0.15	5′ UTR
	cg16586594	16	8806569	0.0005	−0.18	0.0005	−0.19	5′ UTR
	cg08834902	16	8806690	0.0047	−0.23	0.0047	−0.16	5′ UTR
SLC1A6 Chr19p13	cg12695707	19	15121333	0.0002	−0.27	0.0005	−0.16	TSS1500
	cg09470638	19	15121509	0.0010	−0.25	0.0048	−0.14	TSS200
	cg02489552	19	15121531	0.0002	−0.14	0.0002	−0.21	TSS200
SOX18 Chr20q13	cg02231404	20	62679635	0.0010	−0.20	0.0010	−0.21	Body
	cg22138735	20	62679713	0.0005	−0.17	0.0005	−0.17	Body
TEPP Chr16q21	cg04370442	16	58019866	0.0047	−0.15	0.0075	−0.16	Body
	cg12499872	16	58019893	0.0005	−0.31	0.0005	−0.15	Body
CNTNAP1 Chr17q21	cg16308533	17	40838983	0.0005	−0.17	0.0010	−0.19	Body
	cg11629889	17	40839022	0.0002	−0.14	0.0010	−0.16	Body
SNED1 Chr2q37	cg23491743	2	241989271	0.0017	−0.16	0.0030	−0.17	Body
	cg25241559	2	241989379	0.0005	−0.15	0.0005	−0.17	Body

Selection is performed after analysis with two pipelines and application of three selection criteria: (i) absolute *β* value difference >0.10; (ii) *P* value <0.05; and (iii) presence of multiple CpGs per locus. Nucleotide positions accord to NCBI build 37/hg19. Gene group is defined relative to the nearest open reading frame: within 1500 (TSS1500) or 200 bp (TSS200) of a transcription start site, in the 5′ untranslated region (5′ UTR), the first exon of a transcript (exon), in the body of gene (body) or the 3′ UTR (3′ UTR). These six genes were selected for the validation study using Sequenom EpiTYPER

*β diff β* difference, *Chr* chromosome, *Illumina ID* identification according to HM450k, *MMC* myelomeningocele

### Validation study in larger MMC and control cohorts

**Fig. 2:**
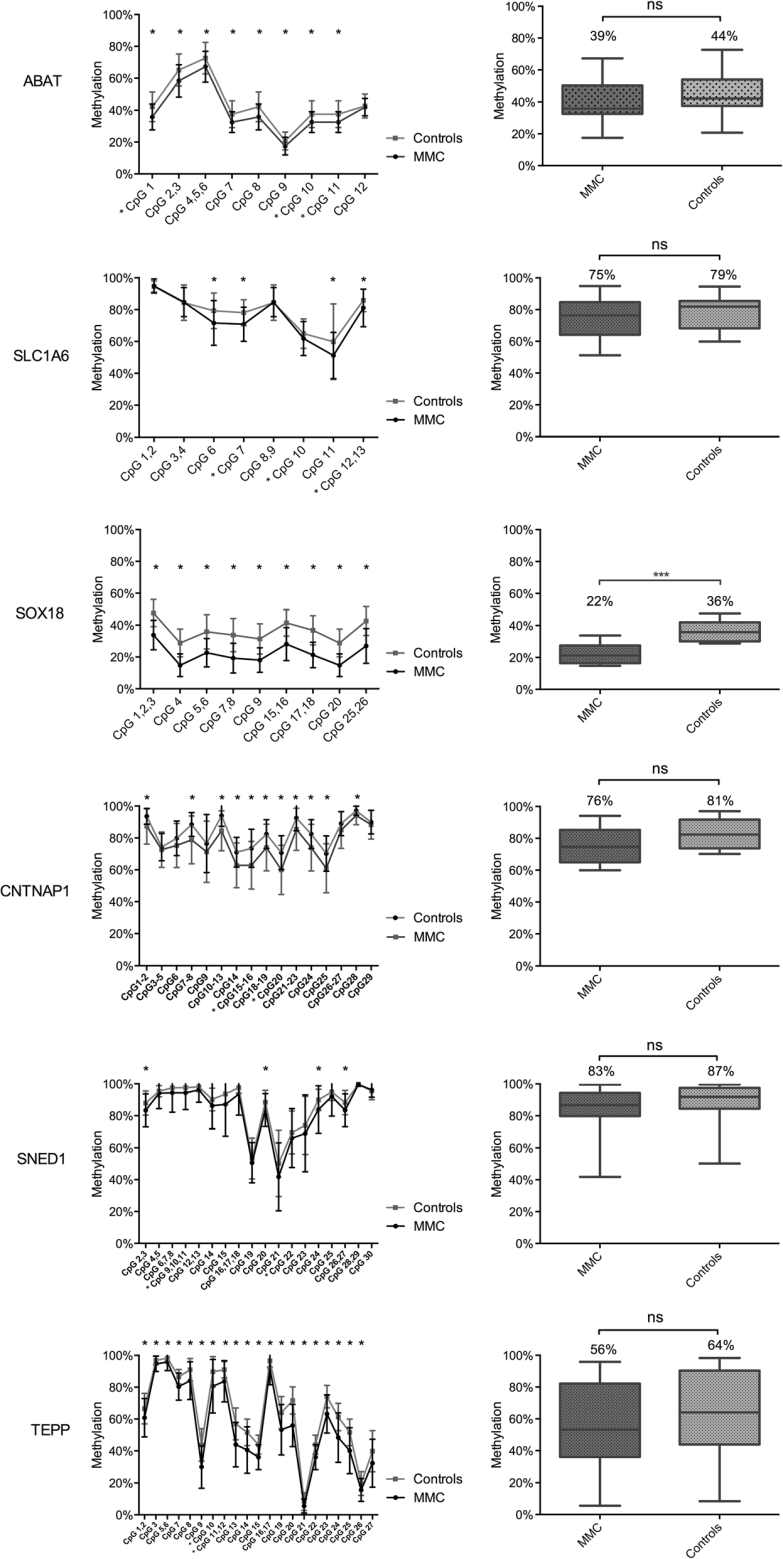
Validation of the top six differentially methylated genes by Sequenom EpiTYPER in MMC patients versus controls. *Left*: methylation pattern for each CpG unit within the amplicons. Multiple *t* test was performed for each CpG. *P value <0.05. *Right*: boxplot representing methylation pattern with box = 25th and 75th percentiles; *bars* = min and max values. The mean methylation level of each group is shown above the plot. The validation study is performed for 83 MMC patients and 30 controls. *CpGs were first identified by HM450k

A validation study was performed with the Sequenom EpiTYPER to quantify DNA methylation for loci that contain the CpGs of the six highest ranked genes. The validation was performed for 83 MMC patients (Additional file 5: Table S6 and previously described in detail in [11]) and 30 unrelated healthy controls. We found for all six genes CpG-specific methylation differences between MMC patients and controls (Fig. 2; left panels, Additional file 5: Table S6). However, when the total methylation value for each gene locus was quantified, a significant change was only detected for the *SOX18* locus between MMC patients and controls (−14 %; 95 % CI (−8 %, −20 %), *P* value = 0.0003) (Fig. 2; right panels). For the other five genes, *ABAT*, *CNTNAP1*, *SLC1A6*, *SNED1*, and *TEPP*, the overall methylation of the studied locus remained not significant. Global DNA hypomethylation has been associated with the *MTHFR 677C>T* variant. Similar to our previous findings for the *HOXB7* locus [11], we did not find an association between *MTHFR 677 CC* versus *CT+TT* genotype and changes in *SOX18* methylation values (Additional file 3: Figure S3). This suggests that an intrinsic defect in the folic acid pathway related to *MTHFR* activity seems not to be involved.

### Chemically induced demethylation versus gene expression analysis

**Fig. 3:**
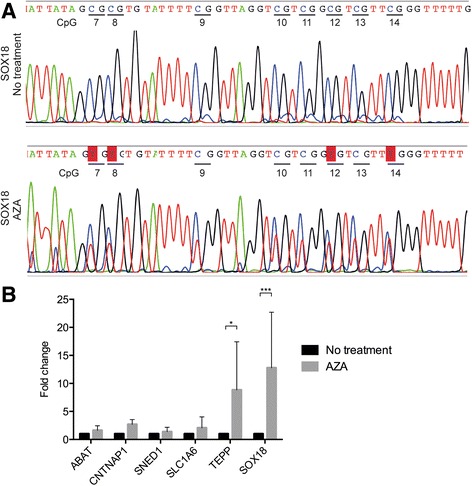
Demethylation studies and gene expression analysis. Demethylation studies were performed using 5 μM 5-aza-2′-deoxycytidine in HEK cell line. **a** Sanger sequencing showing demethylation of *SOX18* after 5-aza-2′-deoxycytidine treatment. The CpGs from the *SOX18* amplicon are annotated below the sequence. **b** Gene expression analysis of top six genes using qRT-PCR. *Standard* standard culture conditions, *AZA* 5-aza-2′-deoxycytidine. *P value <0.05, ***P value <0.001

Demethylation studies were performed in HEK cells that were treated for 72 h with 5 μM 5-aza-2′-deoxycytidine (AZA). Bisulfite sequencing using primers similar to the Sequenom EpiTYPER study (Additional file 5: Table S8) confirmed CpG demethylation for all CpGs within the top six ranked genes (*ABAT*, *CNTNAP1*, *SLC1A6*, *SNED1*, *SOX18*, and *TEPP*) after AZA treatment but again with the most pronounced difference detected for the *SOX18* locus (Fig. 3a). Gene expression was measured for *ABAT*, *CNTNAP1*, *SLC1A6*, *SNED1*, *SOX18*, and *TEPP* using qRT-PCR, but only *TEPP* and *SOX18* expression was significantly increased after AZA treatment (Fig. 3b).

### Gene expression studies in zebrafish

**Fig. 4:**
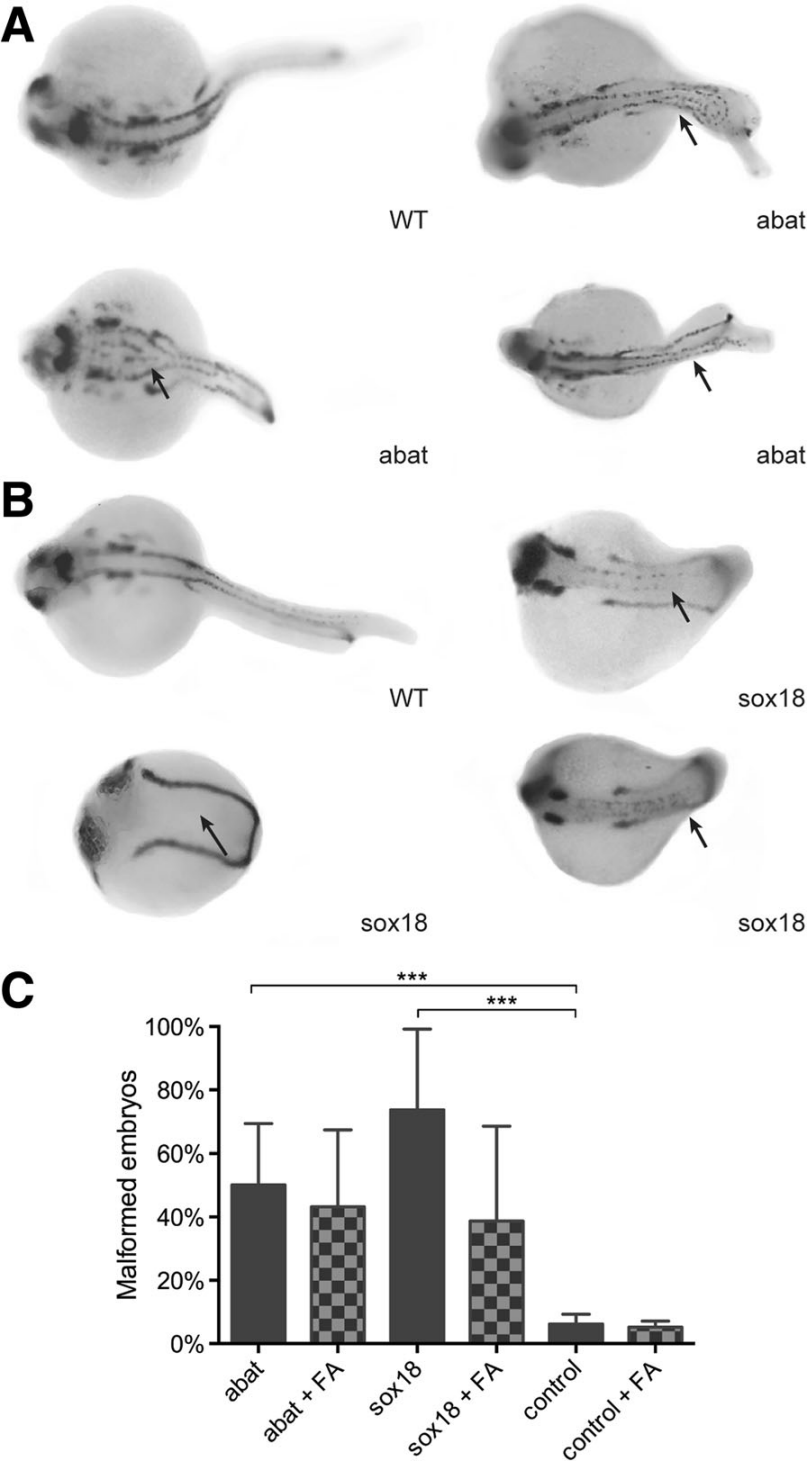
Phenotype analysis of gene overexpression in zebrafish embryos. *Pax2a* staining after microinjection of *abat* mRNA (**a**) and *sox18* mRNA (**b**). Wild-type (WT) zebrafish show expression in the hindbrain, hindbrain-midbrain boundary, neural tube, mesoderm, optic stalk, otic vesicle, and pronephric duct. Spinal cord malformation is indicated with an *arrow*. **c** Phenotype analysis after *pax2a* staining at 24 hpf resulted in respectively 50 and 74 % embryos with an affected phenotype after *abat* and *sox18* overexpression. Folic acid supplementation after gene overexpression did not significantly influence the phenotype. ***P value <0.001

Functional genetic studies were performed in zebrafish to study alterations of *sox18* and *abat* expression during embryogenesis and neural tube formation. Both genes are highly conserved between humans and zebrafish (68 % for *sox18* and 70 % for *abat*). Embryos injected with *abat* mRNA developed abnormal neural tube structures in about 50 % of the embryos though they were only mildly malformed (Fig. 4a). Injection of *sox18* mRNA resulted in a developmental delay with severe malformations in 74 % of the embryos that clearly show abnormal neural tube formation (Fig. 4b). Folic acid could not rescue these neural tube deformities (Fig. 4c).

### SOX18 methylation studies in five MMC patients and their non-affected siblings and parents

**Fig. 5:**
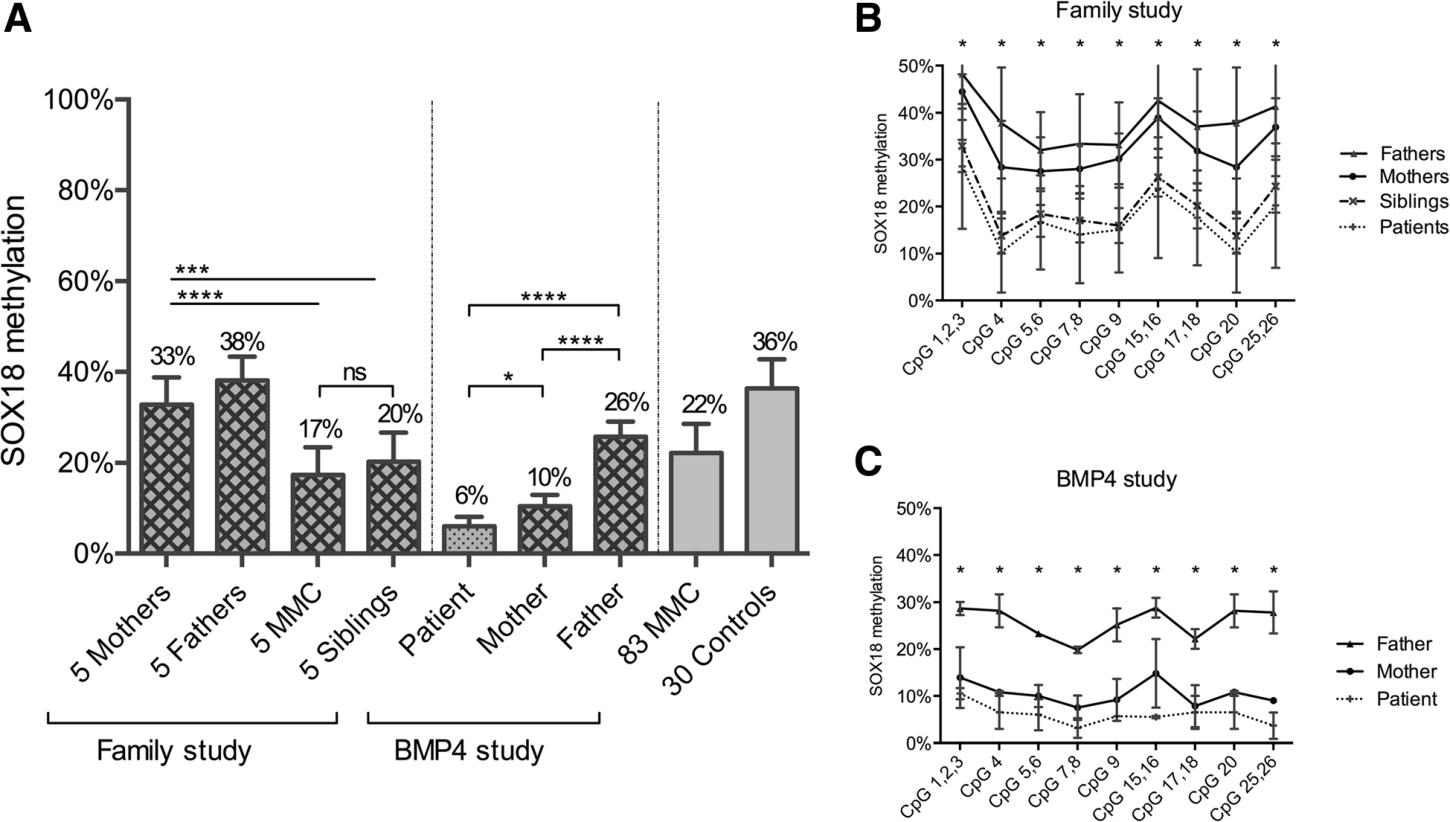
*SOX18* methylation for the family study and the *BMP4* study by Sequenom EpiTYPER. **a** Mean methylation and standard deviation of *SOX18* methylation for the family study and the *BMP4* study. The mean methylation level of each group is shown above the plot. *P value <0.05, **P value <0.01, ***P value <0.001, ****P value <0.0001. **b** Methylation pattern for each CpG unit within the *SOX18* amplicon for the family study. Multiple *t* test was performed for each CpG. *P value <0.05. **c** Methylation pattern for each CpG unit within the *SOX18* amplicon for the *BMP4* study. Multiple *t* test was performed for each CpG. *P value <0.05. The family study consists of MMC patients, their unaffected siblings, and their parents (*n* = 5 for each group). The *BMP4* study describes the family of a patient with a *BMP4* deletion. The father is carrier of the *BMP4* deletion

To study the inheritance of the *SOX18* methylation changes, we quantified *SOX18* methylation for five MMC patients and their non-affected siblings and parents with the Sequenom EpiTYPER (Fig. 5a). Interestingly, not only the MMC patients but also their non-affected healthy siblings presented with similar levels of *SOX18* hypomethylation whereas their parents had methylation values comparable to these of the unrelated control cohort (Fig. 5a). There were no significant DNA methylation differences between the five MMC patients, their siblings, and the overall MMC cohort. Also, the quantification of DNA methylation for the separate CpGs revealed higher methylation values for the parents compared to the MMC patient and its healthy sibling for all CpGs (Fig. 5b). These data would imply that *SOX18* hypomethylation is not sufficient to cause a NTD.

### SOX18 methylation studies in the parents of a MMC patient with a paternally inherited BMP4 deletion

To investigate the influence of an underlying genetic factor, *SOX18* DNA methylation was quantified in a boy with lumbosacral MMC and his parents. The boy has paternally inherited microdeletions that comprise 14q22.1q22.2 and 2p11.2. 14q22.1q22.2 covering the genes *FERMT2*, *DDHD1*, *BMP4*, *DKN3*, and *CNIH* (Additional file 4: Figure S4) while the other deletion in the 2p11.2 region is located in a gene desert region. The father has postaxial polydactyly, severe myopia, and pro-optosis but no spina bifida. His clinical phenotype is compatible with a *BMP4* deletion syndrome. Interestingly, *BMP4* is one of the 45 genes that showed DNA methylation changes in our HM450k analysis (Additional file 5: Table S3) and is also present in the functional enrichment network very closely connected to *SOX18* (Fig. 1a). The *BMP4* deletion patient and also his mother presented significantly lower methylation values compared to the father and the group of unrelated healthy controls (Fig. 5a, b).

## Discussion

Current research supports the hypothesis that abnormal DNA methylation contributes to NTDs [5, 19]. However, rather than DNA hypomethylation of individual candidate loci, a complex combination of environmental and (epi)genetic factors are likely causative for NTDs [20, 21]. Analysis of HM450k methylation data for ten MMC patients and six unrelated healthy controls showed no statistically significant difference in global DNA methylation as determined via the LINE-1 and LINE-2 methylation. However, unsupervised hierarchical clustering showed similar global DNA methylation patterns for the MMC patients. Genes implicated in the folic acid metabolism were not significantly differentially methylated between patients and controls. To investigate which genes are significantly differentially methylated in patients with NTDs, we next analyzed the HM450k data without prioritizing for candidate genes. As the two Infinium assays of the HM450k generate two different types of data [22], we used two analysis pipelines with a different correction and normalization method (WateRmelon and IMA). We identified significant hypomethylation for the genes *ABAT*, *CNTNAP1*, *SLC1A6*, *SNED1*, *SOX18*, and *TEPP*. Further validation by Sequenom EpiTYPER confirmed CpG-specific DNA hypomethylation for these genes. However, only the *SOX18* locus showed highly significant overall hypomethylation for MMC patients. Recessive and dominant mutations in *SOX18* in humans are known to cause hypotrichosis-lymphedema-telangiectasia syndrome with a combination of hair and cardiovascular anomalies, including symptoms of lymphatic dysfunction [23]. Most studies available to date have focused on the role of *SOX18* depletion or loss of function mutants while our study points to a potential role of *SOX18* hypomethylation leading to increased *SOX18* expression. Interestingly, a recent study of Petrovic et al. suggests that Hedgehog signalling up-regulates *SOX18* expression, which promotes migration [24]. Hedgehog signalling is known to play an important role in neural tube development [24, 25]. Evidence for a role for *SOX18* in neural tube formation is also supported by our zebrafish studies that showed strongly malformed tails with abnormal neural tube development in embryos with high levels of *sox18* after injection of its mRNA.

As periconceptional folate is important for NTD prevention, we compared our findings with a large dataset that examined the association between maternal plasma folate during pregnancy and genome-wide DNA methylation in newborn cord blood [14]. Interestingly, one of the top differentially methylated CpGs of this study (cg12695707 within the *SLC1A6* gene locus) was also detected as a hypomethylated CpG in our study (Table 1). Therefore, *SLC1A6* seems to be sensitive to maternal plasma folate in both healthy controls as in MMC patients. In addition, multiple developmental processes seem to be influenced by maternal folate, including neural tube development. Recently, we described the importance of *HOX* gene methylation in neurulation [11], which is also influenced by maternal plasma folate [13]. In addition, we checked if our candidate genes are also represented in another large-scale meta-analysis that investigated the influence of maternal smoking on DNA methylation in newborns [26]. Five of our 75 candidate loci with abnormal methylation being *BMP4*, *ENAH*, *GALNT2*, and *NRP2* and one CpG in a locus without an annotated gene seem to be highly significantly influenced by maternal smoking. Bone morphogenetic protein 4 (*BMP4*) is of special interest as it is involved in craniofacial development and an important candidate gene for cleft palate defects [27]. However, *BMP4* has not yet been implicated directly in lumbosacral MMC.

In addition to dietary and environmental factors, DNA sequence variability influences DNA methylation. The influence of DNA sequence variability on DNA methylation is estimated to differ between 22 and 80 % [28]. In order to investigate how genetic factors might influence DNA methylation, we first genotyped the *MTHFR 677C>T* variant as this variant is known to lead to hyperhomocysteinemia and global DNA hypomethylation especially under low folate conditions [29]. We did not find an additive effect of the *MTHFR* variant on *SOX18* methylation. To investigate whether candidate genes from literature might be differentially methylated, we compared the findings of our top 45 significantly differentially methylated genes with the gene list from a recent NTD wiki database that provides an online up-to-date list of genes implicated in neural tube closure (http://ntdwiki.wikispaces.com). Only one of our top genes, *ENAH*, was present in the candidate gene list. However, the interplay between genetics and epigenetics in driving a disease phenotype is not well understood and is completely unknown for NTD etiology. As we hypothesized that there may be a maternal factor that contributes to a disturbed DNA methylation cycle, we performed *SOX18* methylation studies for five families including the MMC patient and their non-affected parents and sibling. Both parents had methylation levels comparable to these of the control cohort while the MMC patients and their healthy siblings had significantly lower *SOX18* methylation. These findings suggest that there might be a parental genetic or epigenetic contribution that leads to a disturbed one-carbon metabolism and methylation pattern. In addition, we report here the first MMC case with a combined genetic and DNA methylation defect involving a paternally inherited chromosomal deletion that includes *BMP4* and a maternally inherited defect in *SOX18* methylation. The MMC child, born from Caucasian non-related parents, was diagnosed with a lumbosacral NTD and carries a paternally inherited 14q22.1-2 deletion that involves *FERMT2*, *DDHD1*, *BMP4*, *DKN3*, and *CNIH. BMP4* deletions have been associated with variable defects of the eyes, palate, limbs, and brain and with developmental and growth delay but not with NTDs [30, 31]. Mutations in *DDHD1* are found in patients with hereditary spastic paraplegia [32]; *CDKN3* is linked with different cancers while for *FERMT2* and *CNIH*, no human diseases have been described. The father with the same chromosomal deletion has postaxial polydactyly, severe myopia, and pro-optosis but no NTD. Interestingly, methylation studies in the parents and patient showed pronounced hypomethylation of *SOX18* in the mother and patient. Though further biological studies are needed to support any functional interplay between *BMP4* and *SOX18*, this pedigree illustrates the inheritance of a genetic deletion that could cause a NTD in combination with DNA hypomethylation. It is worth to notice that both *SOX18* [24, 33, 34] and *BMP4* [35, 36] have been described as regulators of angiogenesis and this could explain an old concept that there might be a vascular basis for NTDs [37]. The vascular hypothesis states that NTDs result from a disturbance in the timely development of the vasculature. A constant nutrition supply is essential for normal embryonic development. The transition from an avascular to a vascular organism is normally accomplished during the fourth postconceptional week. The neural system has a very prolific growth during the prevascular stage and is therefore the first to outgrow its nutrient supply. As both *SOX18* and *BMP4* are implicated in the establishment of a vascular network, conjunct dysfunction might disturb nutrient supply in early development and lead to NTDs [33, 34].

An important shortcoming of our study is the fact that we only had access to leukocyte DNA for assessment of methylation. We do not know whether differential methylation of the same gene set would be observed in brain or spinal cord tissue of MMC patients. But concordant methylation alterations in brain and blood suggest that blood methylation might be representative for brain methylation [38–42]. Hannon et al. generated an online tool which allows to investigate the correlation of DNA methylation of blood and four brain regions for all probes present on the HM450k [43]. Comparison for *SOX18* methylation shows a good correlation between variation in blood and the cortical regions of the brain. Moreover, it is known that DNA methylation in blood is significantly more variable than DNA methylation in brain tissues [39]. Horvath et al. [41] and Farré et al. [39] identified a DNA methylation signature of age that is not related to cell type composition and that does not require a correction for cellular heterogeneity. This is an interesting finding, as until now there was still no clear evidence that DNA methylation marks of different cell types respond in a similar way to environmental influences. The sample size of our genome-wide study was small, but the reproducibility of significant hypomethylation of the top CpGs in a much larger cohort suggests that the application of two pipelines to analyze the HM450k dataset was a good strategy.

## Conclusions

This is the first study that investigates genome-wide DNA methylation in leukocytes in patients with NTDs. We report *SOX18* as novel risk gene for NTDs but our findings also suggest that *SOX18* hypomethylation and increased expression must interplay with other environmental and (epi)genetic factors that are causative for NTDs. Therefore, further studies should focus on a gene discovery design for MMC that includes both DNA methylation and next-generation sequencing approaches. It is possible that gene variants in combination with changes in methylation are more prone to result in a multifactorial disease as spina bifida.

## Methods

### Description of MMC patients, related healthy siblings, and unrelated healthy controls

A total of 85 MMC patients, 12 healthy related siblings, and 30 age- and gender-matched non-related healthy control subjects enrolled in this study. The MMC patients are followed at the Pediatric Neurology Department of the University Hospital Leuven (all <18 years). Detailed clinical and general characteristics for all these subjects are reported in Additional file 5: Table S6 and have been previously reported in Table 1 of [11]. As sensory and motor functions at and below the level of the spinal cord defect are impaired, paralysis and bowel and bladder dysfunction are present in most of the patients. Folic acid supplementation was recommended, but red blood cell folate was not measured during pregnancy. For the family study, we included the parents of five sibling pairs (pairs 5, 27, 29, 41, and 60 from [11]).

### Description of a case of Caucasian boy with BMP4 deletion

The male patient was born at full term to a G2P1 Caucasian mother by a lower segment caesarean section after an uncomplicated pregnancy with normal prenatal ultrasounds. At birth, he presented an open lumbosacral myelomeningocoele and bilateral talipes equinovarus. The anterior fontanelle was full and bulging. Bilateral lower limb weakness was evident, and initially, he did not have neurogenic bladder or bowel dysfunction. Cranial ultrasound revealed hydrocephaly and Arnold-Chiari II malformation. External ventriculoperitoneal drainage and surgical repair of the spinal defect were performed at the day of birth. Renal ultrasound showed a multicystic dysplastic kidney on the right side.

Comparative genomic hybridization using an 180k oligo array platform (180K Cytosure ISCA v2, OGT, Oxford, UK) showed a 1.665 kb deletion on chromosome 14q22.1q22.2 (14:53,267 987-54,933 219; NCBI/hg19, February 2009) and a microdeletion on chromosome 2p11.2 (2:83,380 184-83,915 440; NCBI/hg19, February 2009). The deleted region on chromosome 14 spans the genes *FERMT2*, *DDHD1*, *BMP4*, *DKN3*, and *CNIH* (Additional file 4: Figure S4); the microdeletion on chromosome 2 does not cover any known gene. The father of the patient has postaxial polydactyly, severe myopia, and pro-optosis and carries both deletions. The mother of the patient has a 45X/46XX mosaicism. She consumed periconceptional synthetic folic acid supplementation. The DECIPHER ID of the patient is 288171 (https://decipher.sanger.ac.uk).

### MTHRF 677C>T genotyping

Leukocyte DNA from MMC patients, related healthy siblings, and unrelated healthy controls was screened for the presence of the *MTHFR 677C>T* variant by PCR and restriction digestion as described [44]. The *MTHFR* genotype of the 85 MMC patients and 12 unaffected siblings is previously reported in [11]. Genotyping for the 10 parents of the family study revealed six parents (3M/3F) with a *CT* genotype and four parents (2M/2F) with a *CC* genotype.

### Genome-wide DNA methylation analysis using the *HM450K*

Leukocyte DNA (1 μg) was subjected to bisulfite treatment using the EZ DNA methylation kit using manufacturers standard protocol (Zymo Research, Irvine CA, USA). Subsequently, genome-wide DNA methylation was assessed using Illumina Infinium HumanMethylation 450K BeadChip (Illumina, Inc., California, USA). The HM450k experiment and data preprocessing were performed as described previously [11]. The analysis was conducted using both the Illumina Methylation Analyzer (IMA) package implicated in the open-source statistical environment R and the WateRmelon R-package [16]. The following three filters were applied on the data for the two pipelines to identify the most significant differentially methylated regions between MMC patients and controls: (i) absolute *β* value difference >0.10; (ii) *P* value <0.01; and (iii) presence of multiple CpGs per locus. The data discussed in this publication have been deposited in NCBI’s Gene Expression Omnibus [45] and are accessible through GEO Series accession number GSE81846.

### DNA methylation of LINE elements and folic acid regulatory genes

We analyzed the methylation values for all LINE-1 and LINE-2 probes from the HM450k study. About 20158 CpG probes correspond to 1498690 LINE-1 and LINE-2 elements (Additional file 5: Table S1). We next analyzed 43 genes (including 698 CpG probes) involved in the folic acid and the one carbon metabolism [18] (Additional file 5: Table S2). Cluster analysis was performed within the R statistical environment.

### Functional enrichment analysis

Based on the results from the genome-wide DNA methylation analysis, a list of CpGs corresponding to differentially methylated CpG sites was generated and tested for enrichment of known gene ontology pathways. This is performed by means of a hypergeometric test and an FDR-based multiple testing correction of the obtained *P* values. The cut-off threshold was set at *P* < 0.001. The enrichment analysis was visualized using the open-source plugin Enrichment Map in Cytoscape 3.2.1.

### Methylation of CpGs for six genes using the Sequenom EpiTYPER

Leukocyte DNA (1 μg) was subjected to bisulfite treatment using the MethylDetector™ bisulfite modification kit (Active Motif, Carlsbad CA, USA). Subsequently, DNA methylation analysis of the top six differentially methylated genes was analyzed by Sequenom EpiTYPER (Sequenom, San Diego, CA, USA) as we described in previous research [11, 46, 47]. The primers are described in (Additional file 5: Table S8). Statistical analyses to quantify DNA methylation differences were performed using the Prism 6 software (GraphPad Software Inc., San Diego, CA, USA). Not all DNA methylation values are normally distributed (D’Agostino and Pearson normality test). A two-tailed *t* test with Welch’s correction was used to assess differences in mean DNA methylation levels between cohorts for the amplicons considered as methylation average and for each CpG unit within this amplicon separately. Detailed characteristics of the amplicon positions in UCSC genome browser can be found in the supplementary folder named “UCSC.”

### Gene overexpression in zebrafish

Wild-type AB zebrafish strains were maintained according to standard protocols [48]. Embryos were produced by natural mating and collected and fixed at different stages based on standard morphological criteria [49]. Zebrafish embryos were injected with *abat* and *sox18* mRNA. The production of mRNA was performed as previously reported [11]. Details of transcripts and primers are described in Additional file 5: Table S9. Off-target effects were assessed by injecting with a standard control MO against beta-globin (5′-CCT CTT ACC TCA GTT ACA ATT TAT A 3′). All injected embryos were life-screened at 24 h post-fertilization (hpf) using a Zeiss Lumar V12 (Carl Zeiss Microscopy, Thornwood, NY, USA) and images were captured with a Leica DFC310 FX digital color camera (Leica Microsystems, Wetzlar, Germany). Experiments were performed in triplicate. We also investigated if addition of 0.1 mM folic acid to the egg water would rescue the phenotype [50]. Ethical approval was obtained for these studies.

### Pax2a whole mount in situ hybridization

Whole mount in situ hybridization (WISH) for the paired box gene 2a (*pax2a*) was performed with digoxigenin-labeled antisense riboprobes as previously described [11, 51]. The influence of *abat* and *sox18* mRNA injection on spinal cord and notochord formation was studied using standard morphological criteria [49]. WISH experiments were performed in duplicate.

### Gene expression analysis in HEK293 cells

The human embryonic kidney (HEK) cell line were cultured under standard conditions or pretreated for 72 h with 5 μM 5-aza-2′-deoxycytidine (Sigma Aldrich, Belgium) for demethylation studies. All cells were maintained at 37 °C in a humidified environment with 5 % CO_2_.

Leukocyte DNA (1 μg) was extracted and subjected to bisulfite treatment using the MethylDetector^TM^ bisulfite modification kit (Active Motif, Carlsbad CA, USA) as we described [46, 47]. The primers from the validation study were used for PCR amplification (Additional file 5: Table S8). Subsequently, Sanger sequencing was performed and the methylation ratios were compared before and after 5-aza-2′-deoxycytidine treatment.

Total RNA was extracted from cells using TRIzol (Invitrogen, Ghent, Belgium) reagent, according to the manufacturer’s protocol. cDNA was synthesized using reverse transcriptase (Invitrogen, Ghent, Belgium). Human gene expression was measured using Sybr Green PCR. qRT-PCR reactions were analyzed using an ABI 7000 real-time PCR machine (Life Technologies). Gene expression was quantified via the ΔΔCt method [52] and normalized to glyceraldehyde 3-phosphate dehydrogenase (*GAPDH*) gene expression. Primer sequences are listed in Additional file 5: Table S10.

## Additional files

Additional file 1: Figure S1. Whole genome methylation in MMC patients versus controls. Unsupervised hierarchical clustering analysis of the subgroups. Methylation values for LINE-1 and LINE-2 repetitive elements are extracted from data obtained with the HM450k. Heatmaps represent 3575 randomly selected CpGs (1 % total CpGs). Green and red represent 0 and 1 methylation, respectively. MMC: myelomeningocele. (TIF 10684 kb)
Additional file 2: Figure S2. Heatmap showing methylation of genes involved in the folate and one carbon metabolism in MMC patients. Methylation values of the genes are extracted from data obtained with the HM450k. Green and red represent 0 and 1 methylation, respectively. MMC: myelomeningocele. (TIF 12951 kb)
Additional file 3: Figure S3. Mean methylation of *SOX18* for MMC patients and controls according to *MTHFR 677 C>T* genotype by Sequenom EpiTYPER. Boxplot representing the methylation pattern of MMC patients and controls divided according to *MTHFR 677 C>T* genotype with box = 25th and 75th percentiles; bars = min and max values. The mean methylation level of each group is shown above the plot. (TIFF 337 kb)
Additional file 4: Figure S4. Genetic deletion on Chr 14q22 of Caucasian boy with lumbosacral myelomeningocele. Bar indicates 1.665-kb deletion on chromosome 14q22.1q22.2 (53,267,987-54,933,219) encompassing the genes *FERMT2*, *DDHD1*, *BMP4*, *DKN3*, and *CNIH*. Nucleotide positions accord to NCBI build 37/hg19. (PDF 9 kb)
Additional file 5:
**Table S1–S10.** (XLSX 1175 kb)

## Abbreviations

AZA: 5-aza-2′-deoxycytidine
BMP4: Bone morphogenetic protein 4
DMR: Differentially methylated region
GEO: Gene expression omnibus
HEK: Human embryonic kidney
HM450k: HumanMethylation 450K BeadChip (HM450k)
HOX: Homeobox
IMA: Illumina methylation analyzer
MMC: Myelomeningocele
MTHFR: Methylenetetrahydrofolate reductase
NTDs: Neural tube defects
SOX18: Sex-determining region Y–box 18
WISH: Whole mount in situ hybridization

## References

[1] Au KS, Ashley-Koch A, Northrup H (2010). Epidemiologic and genetic aspects of spina bifida and other neural tube defects. Dev Disabil Res Rev.

[2] Blom HJ, Shaw GM, den Heijer M, Finnell RH (2006). Neural tube defects and folate: case far from closed. Nat Rev Neurosci.

[3] Blom HJ (2009). Folic acid, methylation and neural tube closure in humans. Birth defects res Part A, Clin mol teratology.

[4] van der Put NM, Steegers-Theunissen RP, Frosst P, Trijbels FJ, Eskes TK, van den Heuvel LP (1995). Mutated methylenetetrahydrofolate reductase as a risk factor for spina bifida. Lancet.

[5] Rochtus A, Jansen K, Geet CV, Freson K (2015). Nutri-epigenomic studies related to neural tube defects: does folate affect neural tube closure via changes in DNA methylation?. Mini rev medicinal chem.

[6] Liu Z, Wang Z, Li Y, Ouyang S, Chang H, Zhang T (2012). Association of genomic instability, and the methylation status of imprinted genes and mismatch-repair genes, with neural tube defects. Eur J Hum Genet.

[7] Wu L, Wang L, Shangguan S, Chang S, Wang Z, Lu X (2013). Altered methylation of IGF2 DMR0 is associated with neural tube defects. Mol Cell Biochem.

[8] Tran S, Wang L, Le J, Guan J, Wu L, Zou J (2012). Altered methylation of the DNA repair gene MGMT is associated with neural tube defects. J Mol Neurosci.

[9] Farkas SA, Bottiger AK, Isaksson HS, Finnell RH, Ren A, Nilsson TK (2013). Epigenetic alterations in folate transport genes in placental tissue from fetuses with neural tube defects and in leukocytes from subjects with hyperhomocysteinemia. Epigenetics.

[10] Wang L, Wang F, Guan J, Le J, Wu L, Zou J (2010). Relation between hypomethylation of long interspersed nucleotide elements and risk of neural tube defects. Am J Clin Nutr.

[11] Rochtus A, Izzi B, Vangeel E, Louwette S, Wittevrongel C, Lambrechts D (2015). DNA methylation analysis of Homeobox genes implicates HOXB7 hypomethylation as risk factor for neural tube defects. Epigenetics.

[12] Barber BA, Rastegar M (2010). Epigenetic control of Hox genes during neurogenesis, development, and disease. Anat Anz.

[13] Kok DE, Dhonukshe-Rutten RA, Lute C, Heil SG, Uitterlinden AG, van der Velde N (2015). The effects of long-term daily folic acid and vitamin B12 supplementation on genome-wide DNA methylation in elderly subjects. Clin epigenetics.

[14] Joubert BR, den Dekker HT, Felix JF, Bohlin J, Ligthart S, Beckett E (2016). Maternal plasma folate impacts differential DNA methylation in an epigenome-wide meta-analysis of newborns. Nat Commun.

[15] Wang D, Yan L, Hu Q, Sucheston LE, Higgins MJ, Ambrosone CB (2012). IMA: an R package for high-throughput analysis of Illumina’s 450K Infinium methylation data. Bioinformatics.

[16] Touleimat N, Tost J (2012). Complete pipeline for Infinium((R)) Human Methylation 450K BeadChip data processing using subset quantile normalization for accurate DNA methylation estimation. Epigenomics.

[17] Price EM, Cotton AM, Penaherrera MS, McFadden DE, Kobor MS, Robinson W (2012). Different measures of “genome-wide” DNA methylation exhibit unique properties in placental and somatic tissues. Epigenetics.

[18] Greene ND, Stanier P, Copp AJ (2009). Genetics of human neural tube defects. Hum Mol Genet.

[19] Greene NDE, Stanier P, Moore GE (2011). The emerging role of epigenetic mechanisms in the aetiology of neural tube defects. Epigenetics.

[20] Wilde JJ, Petersen JR, Niswander L (2014). Genetic, epigenetic, and environmental contributions to neural tube closure. Annu Rev Genet.

[21] Greene ND, Copp AJ (2014). Neural tube defects. Annu Rev Neurosci.

[22] Dedeurwaerder S, Defrance M, Calonne E, Denis H, Sotiriou C, Fuks F (2011). Evaluation of the Infinium Methylation 450K technology. Epigenomics.

[23] Irrthum A, Devriendt K, Chitayat D, Matthijs G, Glade C, Steijlen PM (2003). Mutations in the transcription factor gene SOX18 underlie recessive and dominant forms of hypotrichosis-lymphedema-telangiectasia. Am J Hum Genet.

[24] Petrovic I, Milivojevic M, Popovic J, Schwirtlich M, Rankovic B, Stevanovic M (2015). SOX18 is a novel target gene of hedgehog signaling in cervical carcinoma cell lines. PLoS One.

[25] Lu XL, Wang L, Chang SY, Shangguan SF, Wang Z, Wu LH (2015). Sonic hedgehog signaling affected by promoter hypermethylation induces aberrant Gli2 expression in spina bifida. Mol Neurobiol.

[26] Joubert BR, Felix JF, Yousefi P, Bakulski KM, Just AC, Breton C (2016). DNA methylation in newborns and maternal smoking in pregnancy: genome-wide consortium meta-analysis. Am J Hum Genet.

[27] Chen Q, Wang H, Schwender H, Zhang T, Hetmanski JB, Chou YH (2014). Joint testing of genotypic and gene-environment interaction identified novel association for BMP4 with non-syndromic CL/P in an Asian population using data from an International Cleft Consortium. PLoS One.

[28] Gertz J, Varley KE, Reddy TE, Bowling KM, Pauli F, Parker SL (2011). Analysis of DNA methylation in a three-generation family reveals widespread genetic influence on epigenetic regulation. PLoS Genet.

[29] Friso S, Choi SW, Girelli D, Mason JB, Dolnikowski GG, Bagley PJ (2002). A common mutation in the 5,10-methylenetetrahydrofolate reductase gene affects genomic DNA methylation through an interaction with folate status. Proc Natl Acad Sci U S A.

[30] Bakrania P, Efthymiou M, Klein JC, Salt A, Bunyan DJ, Wyatt A (2008). Mutations in BMP4 cause eye, brain, and digit developmental anomalies: overlap between the BMP4 and hedgehog signaling pathways. Am J Hum Genet.

[31] Lumaka A, Van Hole C, Casteels I, Ortibus E, De Wolf V, Vermeesch JR (2012). Variability in expression of a familial 2.79 Mb microdeletion in chromosome 14q22.1-22.2. Am J Med Genet A.

[32] Tesson C, Nawara M, Salih MA, Rossignol R, Zaki MS, Al Balwi M (2012). Alteration of fatty-acid-metabolizing enzymes affects mitochondrial form and function in hereditary spastic paraplegia. Am J Hum Genet.

[33] Cermenati S, Moleri S, Cimbro S, Corti P, Del Giacco L, Amodeo R (2008). Sox18 and Sox7 play redundant roles in vascular development. Blood.

[34] Matsui T, Kanai-Azuma M, Hara K, Matoba S, Hiramatsu R, Kawakami H (2006). Redundant roles of Sox17 and Sox18 in postnatal angiogenesis in mice. J Cell Sci.

[35] Boyd NL, Dhara SK, Rekaya R, Godbey EA, Hasneen K, Rao RR (2007). BMP4 promotes formation of primitive vascular networks in human embryonic stem cell-derived embryoid bodies. Exp Biol Med.

[36] Rothhammer T, Bataille F, Spruss T, Eissner G, Bosserhoff AK (2007). Functional implication of BMP4 expression on angiogenesis in malignant melanoma. Oncogene.

[37] Stevenson RE, Kelly JC, Aylsworth AS, Phelan MC (1987). Vascular basis for neural tube defects: a hypothesis. Pediatrics.

[38] Masliah E, Dumaop W, Galasko D, Desplats P (2013). Distinctive patterns of DNA methylation associated with Parkinson disease: identification of concordant epigenetic changes in brain and peripheral blood leukocytes. Epigenetics.

[39] Farre P, Jones MJ, Meaney MJ, Emberly E, Turecki G, Kobor MS (2015). Concordant and discordant DNA methylation signatures of aging in human blood and brain. Epigenetics chromatin.

[40] Tylee DS, Kawaguchi DM, Glatt SJ (2013). On the outside, looking in: a review and evaluation of the comparability of blood and brain “-omes”. Am j med genetics Part B, Neuropsychiatric genetics.

[41] Horvath S, Zhang Y, Langfelder P, Kahn RS, Boks MP, van Eijk K (2012). Aging effects on DNA methylation modules in human brain and blood tissue. Genome Biol.

[42] van den Oord EJ, Clark SL, Xie LY, Shabalin AA, Dozmorov MG, Kumar G (2016). A whole methylome CpG-SNP association study of psychosis in blood and brain tissue. Schizophr Bull.

[43] Hannon E, Lunnon K, Schalkwyk L, Mill J (2015). Interindividual methylomic variation across blood, cortex, and cerebellum: implications for epigenetic studies of neurological and neuropsychiatric phenotypes. Epigenetics.

[44] Frosst P, Blom HJ, Milos R, Goyette P, Sheppard CA, Matthews RG (1995). A candidate genetic risk factor for vascular disease: a common mutation in methylenetetrahydrofolate reductase. Nat Genet.

[45] Edgar R, Domrachev M, Lash AE (2002). Gene Expression Omnibus: NCBI gene expression and hybridization array data repository. Nucleic Acids Res.

[46] Izzi B, Decallonne B, Devriendt K, Bouillon R, Vanderschueren D, Levtchenko E (2010). A new approach to imprinting mutation detection in GNAS by Sequenom EpiTYPER system. Clin Chim Acta.

[47] Izzi B, Francois I, Labarque V, Thys C, Wittevrongel C, Devriendt K (2012). Methylation defect in imprinted genes detected in patients with an Albright’s hereditary osteodystrophy like phenotype and platelet Gs hypofunction. PLoS One.

[48] Westerfield M (1995). The zebrafish book.

[49] Kimmel CB, Ballard WW, Kimmel SR, Ullmann B, Schilling TF (1995). Stages of embryonic development of the zebrafish. Dev Dyn.

[50] Ma Y, Wu M, Li D, Li XQ, Li P, Zhao J (2012). Embryonic developmental toxicity of selenite in zebrafish (Danio rerio) and prevention with folic acid. Food Chem Toxicol.

[51] Krauss S, Johansen T, Korzh V, Moens U, Ericson JU, Fjose A (1991). Zebrafish pax[zf-a]: a paired box-containing gene expressed in the neural tube. EMBO J.

[52] Livak KJ, Schmittgen TD (2001). Analysis of relative gene expression data using real-time quantitative PCR and the 2(-Delta Delta C(T)) Method. Methods.

