# Supplementary figures and images for: Methylome analysis for spina bifida shows SOX18 hypomethylation as a risk factor with evidence for a complex (epi)genetic interplay to affect neural tube development
Source: Clin Epigenetics. 2016 Oct 13;8:108. doi: 10.1186/s13148-016-0272-8 (PMC5064967; doi:10.1186/s13148-016-0272-8)

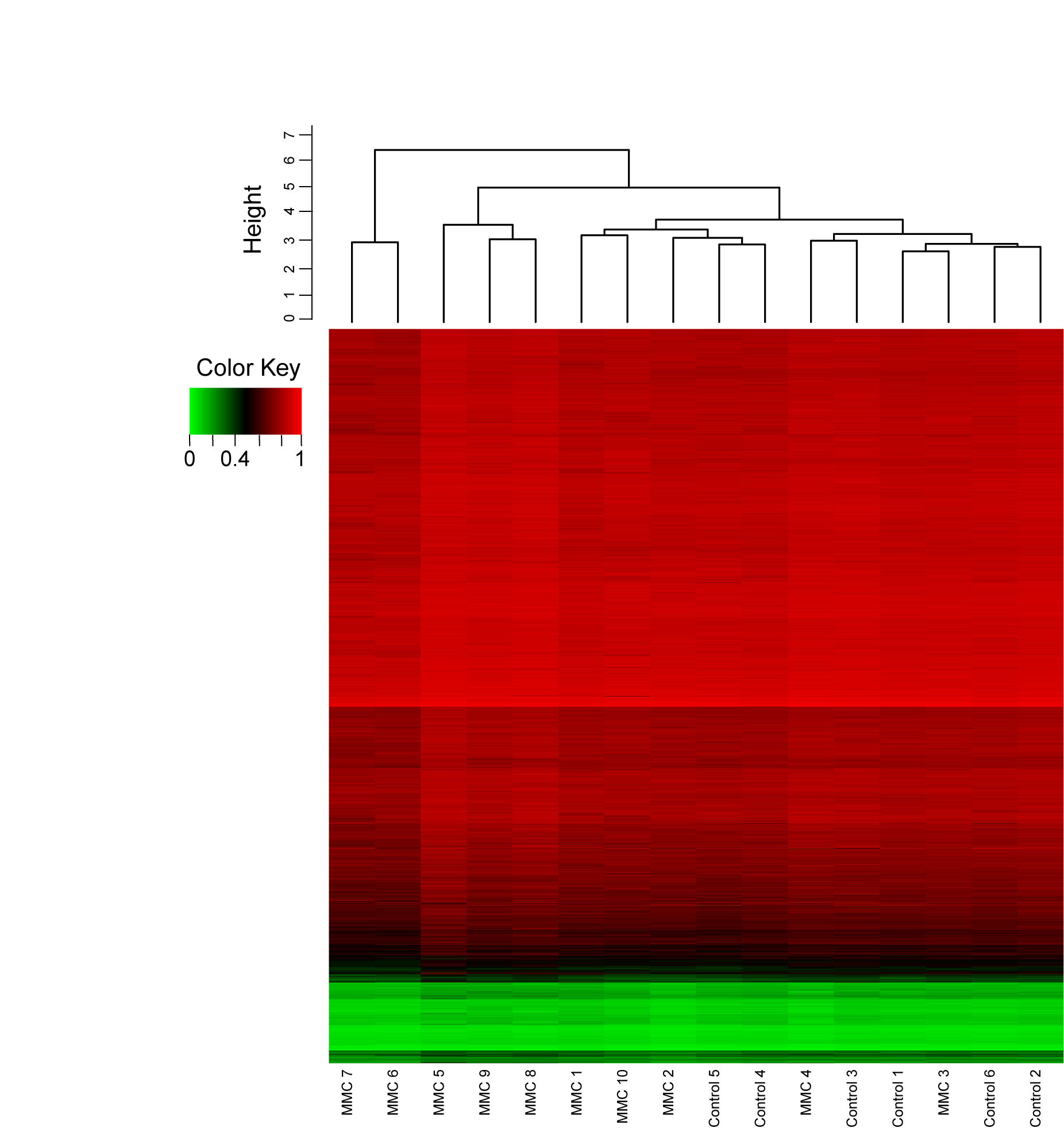

Supplement: Additional file 1: Figure S1. — Whole genome methylation in MMC patients versus controls. Unsupervised hierarchical clustering analysis of the subgroups. Methylation values for LINE-1 and LINE-2 repetitive elements are extracted from data obtained with the HM450k. Heatmaps represent 3575 randomly selected CpGs (1 % total CpGs). Green and red represent 0 and 1 methylation, respectively. MMC: myelomeningocele. (TIF 10684 kb) [file 13148_2016_272_MOESM1_ESM.tif]

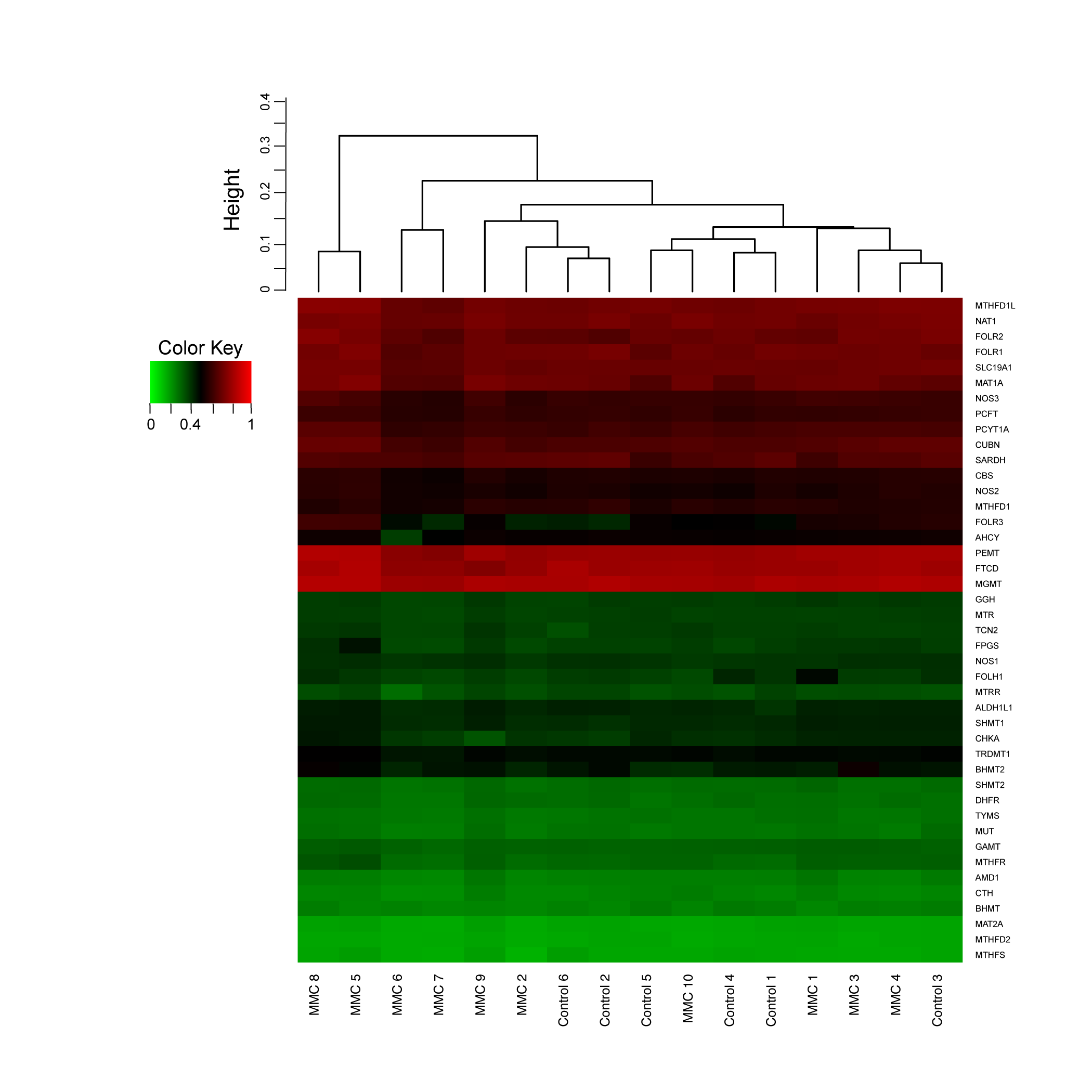

Supplement: Additional file 2: Figure S2. — Heatmap showing methylation of genes involved in the folate and one carbon metabolism in MMC patients. Methylation values of the genes are extracted from data obtained with the HM450k. Green and red represent 0 and 1 methylation, respectively. MMC: myelomeningocele. (TIF 12951 kb) [file 13148_2016_272_MOESM2_ESM.tif]

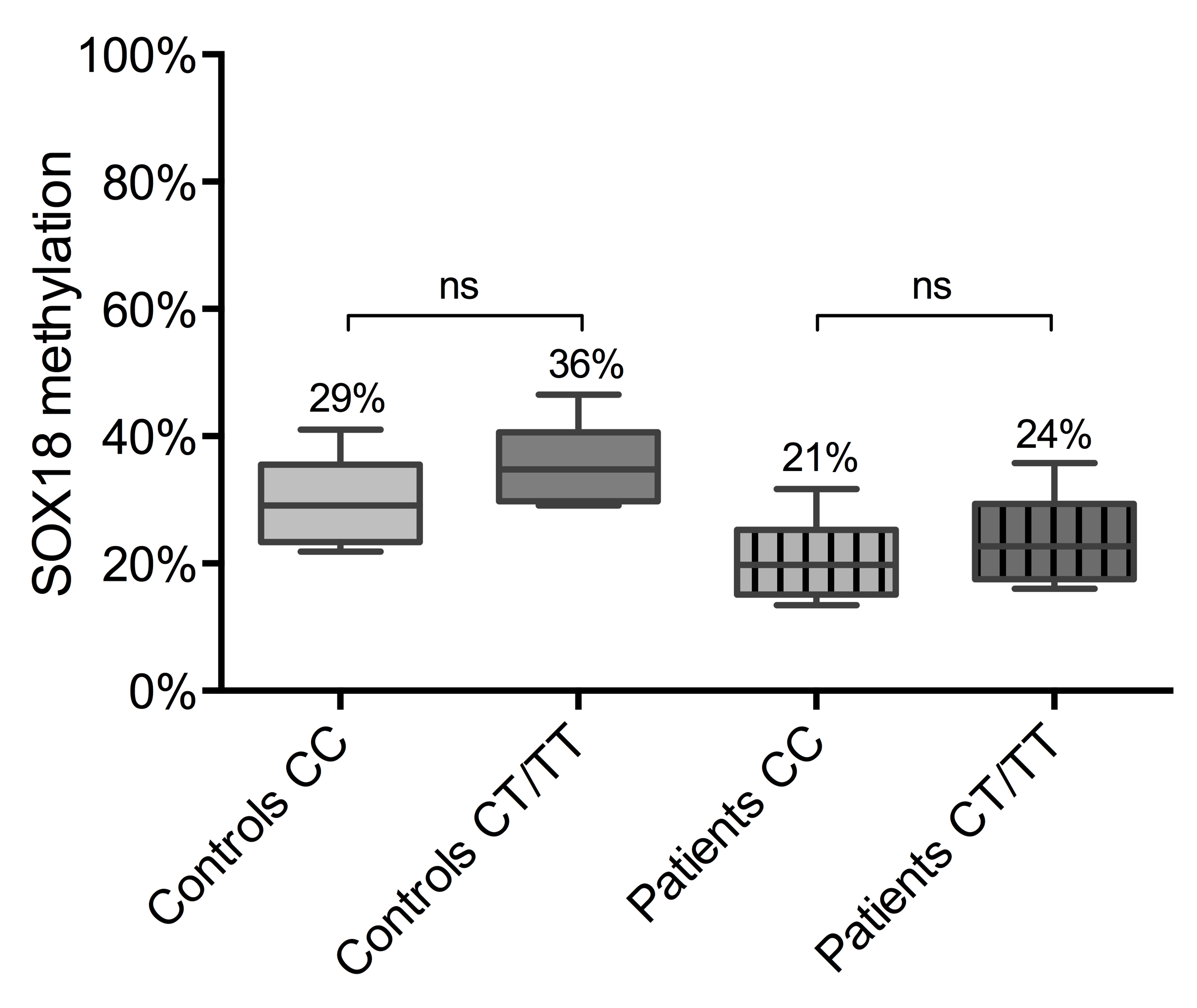

Supplement: Additional file 3: Figure S3. — Mean methylation of SOX18 for MMC patients and controls according to MTHFR 677 C>T genotype by Sequenom EpiTYPER. Boxplot representing the methylation pattern of MMC patients and controls divided according to MTHFR 677 C>T genotype with box = 25th and 75th percentiles; bars = min and max values. The mean methylation level of each group is shown above the plot. (TIFF 337 kb) [file 13148_2016_272_MOESM3_ESM.tiff]

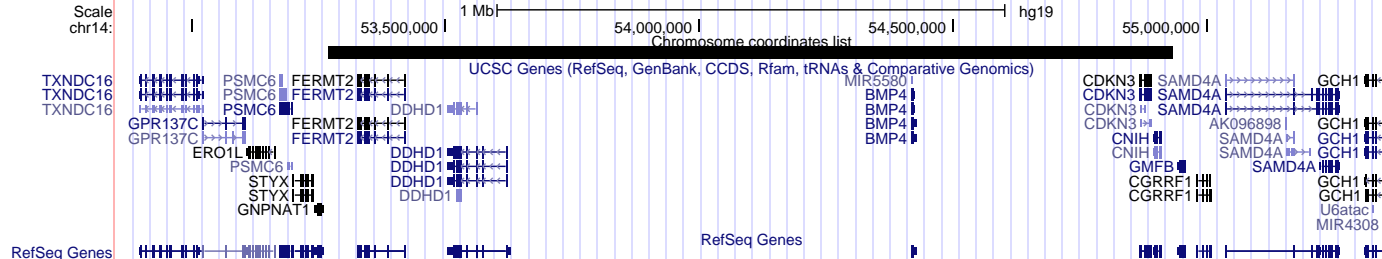

Supplement: Additional file 4: Figure S4. — Genetic deletion on Chr 14q22 of Caucasian boy with lumbosacral myelomeningocele. Bar indicates 1.665-kb deletion on chromosome 14q22.1q22.2 (53,267,987-54,933,219) encompassing the genes FERMT2, DDHD1, BMP4, DKN3, and CNIH. Nucleotide positions accord to NCBI build 37/hg19. (PDF 9 kb) [file 13148_2016_272_MOESM4_ESM.pdf]
